# Challenges Faced by Healthcare Professionals During the COVID-19 Pandemic: A Qualitative Inquiry From Bangladesh

**DOI:** 10.3389/fpubh.2021.647315

**Published:** 2021-08-10

**Authors:** Shaharior Rahman Razu, Tasnuva Yasmin, Taimia Binte Arif, Md. Shahin Islam, Sheikh Mohammed Shariful Islam, Hailay Abrha Gesesew, Paul Ward

**Affiliations:** ^1^Sociology Discipline, Khulna University, Khulna, Bangladesh; ^2^Development Studies Discipline, Khulna University, Khulna, Bangladesh; ^3^Institute for Physical Activity and Nutrition, Deakin University, Melbourne, VIC, Australia; ^4^Discipline of Public Health, College of Medicine and Public Health, Flinders University, Adelaide, SA, Australia; ^5^Epidemiology, School of Health Sciences, Mekelle University, Mekelle, Ethiopia

**Keywords:** COVID-19, health professionals, workload, mental health, Bangladesh

## Abstract

**Background:** The coronavirus disease 2019 (COVID-19) pandemic has caused increasing challenges for healthcare professionals globally. However, there is a dearth of information about these challenges in many developing countries, including Bangladesh. This study aims to explore the challenges faced by healthcare professionals (doctors and nurses) during COVID-19 in Bangladesh.

**Methods:** We conducted qualitative research among healthcare professionals of different hospitals and clinics in Khulna and Dhaka city of Bangladesh from May 2020 to August 2020. We conducted 15 in-depth telephone interviews using a snowball sampling technique. We used an in-depth interview guide as data were collected, audiotaped, and transcribed. The data were analyzed both manually and using QDA Miner software as we used thematic analysis for this study.

**Results:** Seven themes emerged from the study. Participants experienced higher workload, psychological distress, shortage of quality personal protective equipment (PPE), social exclusion/stigmatization, lack of incentives, absence of coordination, and proper management during their service. These healthcare professionals faced difficulty coping with these challenges due to situational and organizational factors. They reported of faith in God and mutual support to be the keys to adapt to adversities. Adequate support to address the difficulties faced by healthcare professionals is necessary for an overall improved health outcome during the pandemic.

**Conclusion:** The findings highlight the common challenges faced by healthcare professionals during the COVID-19 outbreak. This implies the need to support adequate safety kits, protocols, and support for both physical and mental health of the healthcare professionals.

## Introduction

The COVID-19 outbreak was declared as a global pandemic on March 11, 2020 (1). Although social distancing is the most effective way to contain the outspread of this virus, this is not easy to implement for healthcare professionals who require direct contact with COVID-19 patients and puts them under a high risk of being infected themselves (2). Frontline healthcare professionals are particularly vulnerable during this pandemic owing to their commitment to contain the disease (3). As of October 15, 2020, there were around 4,797 COVID-19 cases for doctors and nurses with more than 100 deaths of physicians in Bangladesh (4). Besides physiological threats, such public health emergency affects the psyche of healthcare workers, including professional stress, fear of infection, and feeling helpless (5).

The number of doctors in Bangladesh government healthcare facilities is scarce (5.26 doctors/10,000 people). Hence, many healthcare professionals worked around 17 h, including long tele-counseling shifts each day (6). To mitigate this challenge, the government appointed an additional 2,000 doctors on May 2020 (7). Further, healthcare professionals faced acute shortage of masks, hand gloves, and personal protective equipment (PPE) to protect themselves from COVID-19 infection (8). Moreover, locally produced PPEs, masks, and other kits provided by the authority are being reported to be of low quality and unable to protect the medical workforce from being infected (9).

Healthcare professionals also suffered from insomnia, loneliness, sleep disorder, and mental depression as a result of the workload and related stress (10). They were experiencing anxiety attacks as well as frustration due to a lack of knowledge, environmental changes, and fear of infection both by themselves and by their family members (11). Currently, healthcare professionals are also bound to maintain physical distance from their family members to reduce the risk of contagion, which results in further psychological distress (12). Hence, a special attention to monitor the psychological issues of high-risk population exposed to COVID-19 becomes more essential (13).

When it comes to the challenges faced by the healthcare professionals of Bangladesh during COVID-19 pandemic, concerns raised from bad governance cannot be ignored. The number of PPEs provided by the government was insufficient for healthcare professionals, and they were mostly untrained regarding how to use them. This resulted into an alarming rate of infection among the medical workforce (14). Recent studies emphasize on strengthening the healthcare governance in Bangladesh by properly distributing healthcare facilities between urban and rural areas, public and private facilities, enhancing the role of media, increasing the recruitment of healthcare workers, and concentrating on the provision of necessary healthcare equipment such as intensive care units and oxygen supply (15–17).

Doctors are facing tremendous difficulties at work during the COVID-19 pandemic (18). Despite these obstacles, healthcare professionals have adapted to deal with the prevailing health crisis. A previous study (19) has shown that meditation, relaxation as well as music therapy can help to mitigate the daily stress. During the severe acute respiratory syndrome (SARS) outbreak in 2005, healthcare professionals took some initiatives to cope with the stress associated with the pandemic. The coping mechanisms included avoidance of news about the SARS pandemic, small gatherings after work where problems can be shared as well as participating in other recreational activities (20). Proper training, PPE, and medical assistance are important to support healthcare providers (6); however, these are not available in Bangladesh. A number of studies have been conducted on COVID-19-related issues in Bangladesh; however, there are no qualitative studies on the challenges faced by healthcare professionals during the current COVID-19 pandemic. As qualitative research is known for generating rich information in health research (21), we attempted to address this research gap to get a more in-depth knowledge of the individual experiences, beliefs, opinions, behaviors, and feelings of the healthcare professionals during the pandemic (22).

## Theoretical Framework

We used the stress theories to understand the challenges healthcare professionals in Bangladesh are facing during the COVID-19 pandemic. The COVID-19 outbreak has generally caused public stress (23) as people go through a series of physical and mental challenges both inside and outside which affects their own subjective evaluations (24). Ursin and Eriksen (2004) provide a further explanation on how people go through stress during a crisis. The authors used the term “stress” to denote four different views, namely, “stress stimuli,” “stress experience,” “non-specific general stress response,” and “experience of the stress response” (25). According to Cognitive Activation Theory of Stress (CATS) theory, people acquire knowledge when handling adversities, and a normal, well-balanced stress at such situations should be common. Response to stress is important as this provides the energy that enables them to fight against the odds. However, when there is a disparity between the expected and actual circumstance, the stress response mechanism starts struggling (26). While stress response is essential to face challenges, higher levels of sustained stress can lead to physical and mental disorders. We argue that the sustained workload and mental stress of the healthcare professionals during the pandemic originate an acquired expectancy referred to as “hopelessness”(27).

## Methods

An exploratory qualitative inquiry was employed to understand the in-depth knowledge of challenges dealt by health workers from Khulna and Dhaka city in Bangladesh from May to August 2020. Doctors and nurses who are willing and provided treatment at different hospitals and clinics in Bangladesh during the COVID-19 pandemic participated in this study. We selected 15 respondents for the in-depth interviews through the snowball sampling technique. The participants were recruited through referrals of healthcare professionals from our previous acquaintances. We used this technique as healthcare professionals who were willing to participate in this study were extremely hard to find during the pandemic. The in-depth interview was conducted through telephone. We developed an in-depth interview guide to probe questions for the interview process. The items for the interview guide were generated through searching the relevant literature. Only contents related to the present study were considered, while pieces of pure medical literature were excluded from the review. The guide consisted of questions on barriers related to workload, severity of the illness and associated stress, availability and quality of PPE, COVID-19-related challenges, and coping strategies to manage the barriers.

SRR, TY, TBA, and MSI (academicians who completed their second degree) conducted the interviews and collected data through multiple sessions and with the convenience of the participants. The duration of each session was 30–40 min in general, and the interviews were recorded through an audio recording application/device, which was transcribed in the next stage. We used the follow-up questions to extract rich information during the interviews. Verbal probes such as repeating the ideas and phrases of participants and showing enthusiasm to a particular topic during the interviews were part of the probing strategy. Apart from the authors, two trained research assistants were appointed to manage the data collection and transcription. TBA and MSI independently coded the data from verbatim transcript as the process included the development of a code structure initially. The whole coding procedure was reviewed and finalized with the consent of all authors. We applied a deductive approach suggested by Miles and Huberman (1994) (28) using thematic analysis technique (29). The most recurring and significant quotes were selected to exemplify the predetermined themes. While analyzing, we focused on the meaning, context, phrases, frequency, and intensity of the statements of our participants. We analyzed the data both manually and using QDA Miner (version 5) software. The QDA miner is useful in managing a large volume of qualitative data extending the scope of manual analysis. It is largely used by researchers and experts for conducting qualitative research worldwide.

We maintained standard ethical protocols to conduct this research. The study protocol was approved by the person who blinded for peer review. At the beginning of the interviews, informed consent was sought from each participant after a briefing about purpose of the research was done. The identities of the respondents were kept confidential, and they assured that the information provided by them would only be used for academic research.

## Results

### Characteristics of Study Participants

Fifteen respondents were included in the in-depth interview. The summary of the participants and their details are provided in Table 1.

**Table 1:** Sociodemographic profile of participants.

**Age**	**27–58 years**
Sex	Seven men and eight women
Marital status	All were married except one doctor
Study Area	Khulna (*n* = 9) and Dhaka (*n* = 5)
Institutions	Doctors and nurses from four medical facilities (KMC and GMCH, Khulna and DMCH and JBFH, Dhaka)^*^
Type of Institutions	Two private and two government hospitals
Occupation	Six doctors and nine nurses

*Total participants were 15 (both doctors and nurses)*.

* *KMC, Khulna Medical College (Khulna); GMCH, Gazi Medical College and Hospital (Khulna); DMCH, Dhaka Medical College and Hospital (Dhaka); JBFH, Japan Bangladesh Friendship Hospital (Dhaka)*.

Seven themes emerged from the unstructured interviews, i.e., workload, PPE, social acceptance, mental health, incentives, coping strategies, coordination, and direction of the respondents during the COVID-19 pandemic.

### High Workload

Participants indicated that the health sector faces a shortage of medical workers. Moreover, many registered doctors do no practice medicine, resulting in higher workload by the active medical workforce in public as well as in private facilities. In the private facilities, doctors were usually provided with a 1-day break each week. Doctors were working for long shifts in their working days and during holidays *via* telecommunication. For example, Participant 3 said,

*You are asking the doctors about their workload! When people were busy partying at the eve of Eid-ul-Fitr festival, we were working in the hospital. I had a shift even on Eid day. Moreover, I was diagnosed as COVID-19 positive on 15*^*th*^*of June 2020, which demands for at least a 21 days recovery process after being further tested as being COVID-19. But we cannot afford that luxury as the hospital does not have enough human resources. Consequently, I had to join my work right after accomplishing my recovery from the virus*.

Apart from enduring tremendous physical pressure, excessive workload also leads to increased mental stress. Medical facilities also have few nurses, who had to work 16–17 h shift per day. Additionally, fear of infection prevented workers from joining their workplace. Participant 5 said,

*Since we have completed our nursing degree, so we are supposed to be psychologically well equipped to serve people in any medical emergency. But at the very beginning of the coronavirus outbreak, many of us suffered from a fear of infection and were too afraid to come to work. This decline in the regular number of nurses created too much workload for us*.

Healthcare professionals who were younger and working in Dhaka-based hospitals reported of higher workload in this study. This might be due to a higher work assignment for younger people and a greater outbreak of COVID-19 in the capital city. When asked about workloads, Participant 12 shared with frustration,

*Dhaka is hit most severely during the first wave of COVID-19. It is the capital city of the country with 20 million population and the largest international airport. People are landing here from countries with high infection rate every day and the disease is spreading like bushfire. We are admitting a large number of patients each day and having a really difficult time dealing with it*.

### Lack of PPE

Participants repeatedly pointed out that PPE supplied by their hospitals were either inadequate or of low-quality. Though the government demanded on the mass media that every hospital has been provided with the required numbers of PPEs, the fact on the ground was different. Especially, study participants in private medical facilities need to buy their own PPEs as they were not sure of the availability in the health facilities. Participant 1 corroborated the issue.

*Despite the need to have a regular supply of PPEs, the hospital does not have enough of them in its possession. I have received one PPE per week from Japan Bangladesh Friendship Hospital, which is not sufficient. Consequently, I am needed to buy PPE at my expenses to ensure my safety during work. Another threatening fact regarding PPEs came into my notice from a number of national newspapers. Some corrupt businessmen are generating new PPE's from the ones that have been dumped as medical wasted in Keraniganj, Dhaka. This issue gave me quite a shock and made me question my oath to serve the mass people in any given situation*.

The PPEs provided by the authority were made of plastic-type material. The shortage of PPE also declined to some extent with time. An additional complaint came from the nurses that they had to face acute shortage of PPEs as doctors were the primary focus here and the need for an adequate supply of PPEs for nurses was relatively ignored. Participant 6 noted,

*At the first slot, we were provided with a huge number of poor-quality PPEs which made the pandemic situation more vulnerable for health professionals like us. But as of now (month of June), we have a steady supply of good quality PPEs which can efficiently protect us from this* virus. *From my perception, there is no lack of PPE in the present condition*.

### Low Social Acceptance

Social stigma was another challenge for the healthcare professionals during the COVID-19 pandemic. The neighbors perceived them as a nuisance and usually avoided communication for fear of infection. In some cases, landlords raised monthly house rents of the medical workers and evicted them from their property if they were tested COVID-positive. Sometimes, their maintenance of social distance became rather cruel, and this disturbed the healthcare professionals. Two of the statements represent this condition:

Participant 3: “*Haha! Mass people always perceive us doctors as “butchers” in this country. We are shown some respect over social media posts, but there is no respect for doctors in the real-world. Red flags are used to mark the zone containing COVID positive patients, but from my perspective, these flags are playing the role of barriers. While we need more psychological support from general people, working within the red zone has completely excluded us from society.”*

Participant 5: “*Actually, I feel deeply disturbed when I talk about the issue of social acceptance. When I started serving contagious patients during this pandemic, people of my community treated me in a way which made me feel like I was a raped woman… (Crying). But I have taught myself to endure that pain and work as a frontline fighter against this deadly virus.”*

Parents of healthcare professionals remained concerned about their children working in such a risky environment. They often tried to bargain with them to stay home, but it was merely parental concern, and the participants continued work after pacifying them. Generally, their relatives maintained a social distance and refrained from visiting their houses. But participants considered this as positive to ensure the safety of both their relatives and their family members.

### Mental Health Problems

People working in the medical sector are trained to think and act steadily in any medical emergency. Regardless of that training, participants mentioned that they had to cope with different psychological challenges, including anxiety, depression, insomnia, and fear of sudden death during the COVID-19 pandemic. Participant 2 said,

*Being a doctor has taught me to have full control over my mind. Despite that control, the current pandemic makes me anxious sometimes as many doctors are being infected during their service toward COVID-19 patients. There is one incident worth mentioning in this context. Witnessing the death of patients is part of the job for us, but I had to witness the death of a medical doctor in Sylhet due to COVID-19, which was a first for me. It was the most shocking thing during my lifetime working experience. After this experience, I started having trouble sleeping*.

Healthcare givers serve in an atmosphere where the fear of infection prevails at its largest. Despite that, participants were more concerned about family members being infected by them rather than themselves being infected, leading to further mental stress. Participant 4 mentioned,

*To me, psychological pressure mainly consists of anxiety regarding the safety of my family. I am a widow, and my daughters are dependent on me both economically and for the sustenance of their daily lives. This familial condition puts me in a lot of pressure and forces me to think about what would happen to my daughters if I was diagnosed as a corona virus-positive and died. The constant thought of leaving my daughters all alone in this world is quite stressful*.

Witnessing sudden death of colleagues created a feeling of helplessness among the healthcare professionals, leading to many of them to experience insomnia. The lack of appreciation by colleagues also caused psychological pressure. One of the nurses mentioned that doctors do not appreciate them enough.

*Participant 6: We work with extreme fear and risk of infection risking our lives, but we get no appreciation. People think only doctors are contributing to save lives. We (nurses) are always ignored and underpaid in this country. It's nothing new*.

### Lack of Incentives

All participants were aware that there was no extra-incentive for them despite working extra hours. Some incentives were promised by the government, such as providing treatment cost in case of infection and providing an isolation room to ensure safe inhibition. But none was implemented in the real life. Further, participants strongly believed that these initiatives were not going to be implemented shortly. For example, Participant 3 said,

*Government announced that if anyone got infected by coronavirus during their service, the authority would provide some money for treatment. Surprisingly, I did not receive any monetary support to bear my treatment cost when I was diagnosed as COVID-positive. Their announcement is void as always, and it is never going to be implemented. Though we are getting two basic salaries of around 50,000, which is not enough for us*.

While the incentives provided by the authority for the employees in the government facilities were not satisfactory, the condition of the healthcare professionals working in private facilities was even worse. There was no monetary incentive for the healthcare professionals working in private facilities if they got infected or died during their service. The participants were depressed about this discrimination between public and private employees. Moreover, they were also deprived of basic amenities such as break between work shifts or provision of meals raising frustrations. Participant noted,

*We have seen that roster system is in place to arrange the shifts of the health professionals in the government hospitals. As a result, government doctors get seven days off after completing a seven-day shift with Corona patients. Unfortunately, we, the private clinic workers, do not get any incentive like that. I don't even get my meals from the hospital*.

### Lack of Coordination and Direction

The WHO and government guidelines were changing continuously given the disease is new and previous knowledge is little. Consequently, doctors remained uncertain about the line of treatment. These uncertainties created additional mental stress for medical professionals.

The participants reported that patients were unaware of any safety protocols. COVID-19-positive patients often come to medical facilities to receive standard medical consultation, which put COVID-negative patients as well as the medical workers at-risk. In several cases, doctors and nurses got infected because patients did not reveal that they were COVID-19-infected. A high-level coordination failure was prevalent in the healthcare administrations.

Moreover, healthcare workers were dissatisfied about some discriminatory initiatives taken up by the authority. Participants mentioned the case of the bank sector, where employees worked for only 20 days in April and May. In contrast, healthcare professionals did double or triple shifts, which was frustrating. Besides, they did not have any training regarding how to function correctly in a virus outbreak. It was also perceived that the authority involved more administrators and fewer specialists to tackle down this pandemic. For example, Participant 3 mentioned,

*I want to mention one more issue here. It is needed to create a committee containing doctors as well as virologists who are specialized in providing guidelines in the context of how to handle the current COVID-19 situation in Bangladesh best. Instead, the government has created a task force containing DCs, UNOs and other administrative personnel who possess no knowledge about the virus*.

### Coping Strategies

All of the participants expressed that belief upon God kept them relaxed. Support from family members and colleagues was also an essential coping mechanism. The healthcare professionals maintained regular conversations with colleagues maintaining social distance and tried to be benevolent with each other in their workplace. This supportive environment helped them a great deal in reducing their mental stress. Keeping their sacred oath in mind, they were always more concerned about their patients than their well-being. This concern for the well-being of mass people served as a coping mechanism on its own. For instance, Participant 4 said,

*I cope with the challenges faced in my workplace with the support of my family, colleagues and a firm belief on the almighty's plans for all of us. The support of my close ones and trust in the almighty provides me with a sense of mental strength encourages me to stay positive any crisis. I also take mental notes that this is my job, and I must do it. If I become nervous in performing my duties, then how would the general people survive?*

Apart from taking mental support from friends and families, healthcare professionals tried to follow every medical rule and regulation in their ability to keep safe from infection. The study protocol was approved by the Ethical Clearance Committee of Khulna University. Other participants reported meditation as means to increasing mental strength. Overall, participants put faith in a greater force in this crisis and keep reminding themselves that as they were working for the well-being of humanity.

## Discussion

Our results showed that frontline healthcare professionals in Bangladesh had an increased workload during this crisis and a potential system failure in the healthcare sector. Lack of sufficient healthcare workers, knowledge about the virus, and basic training were some of the reasons leading to excessive workload, which consequently gave rise to psychological stress. This finding is consistent with some of the existing literature (30, 31). A previous study also showed that excessive work pressure was responsible for mental distress, insomnia, physical weakness as well as fear of infection of the healthcare professionals (32). Our study also focused on the lack of quality PPEs prevalent in the healthcare facilities. It was reported that the insufficiency consequently led to an increasing rate of infection among healthcare professionals in Bangladesh. Several studies have found that insufficient PPE triggered the spread of the viruses among healthcare professionals (33, 34). Besides, wearing PPE for a long time was a crucial challenge for participants, subsequently resulting in drinking less water than necessary, which might have affected their immunity (35).

Coordination failure was prevalent among different administration sections in each facility where the respondents worked, resulting in a chaotic environment. Consequently, both doctors and patients were unsure about the protocols needed to maintain safety, which further increased the risk of infection. Insufficiency of medical staff and equipment was common, resulting in excessive workload and safety hazard (36). This workload and constant fear of infection both for themselves and for their family members put participants under substantial psychological stress (11). Social acceptance from neighbors, colleagues, and peer groups could act as a lifeline in removing this psychological stress. But the social reaction of most cases was still adverse toward the medical workforce, and they were shunned from their social life. Hence, the experience of medical professionals was pretty challenging during the pandemic. They still took coping strategies such as putting their faith in God, treating each other with kindness, and soothing conversation with a peer group to cope up with the stress to some extent.

We observed that most of the participants in this study required adequate protective supplies and proper rest, which is consistent with the present study (37). Psychological stress faced by healthcare professionals during public health emergencies included constant worries about infecting children and parents of an individual, fear of death, anxiety about critical patients, and personal danger (38, 39). Healthcare professionals felt anxious when their colleague was infected by COVID-19 (9). We also observed that healthcare professionals who had children were emotionally distressed to maintain distance from their loved ones due to a higher risk of being infected by COVID-19. The finding was similar to another previous research (20). Nurses expressed dissatisfaction with the workload as they are not appreciated enough, although it is evident that they often provide quality healthcare services like the doctors (40).

Healthcare professionals also faced stigma from their neighbors and relatives. Neighbors perceived that the health workers carry a higher risk of infection from their exposure to patients. As a result, healthcare professionals were shunned from society and treated harshly, which sometimes demotivated them to serve patients. However, previous study documented that healthcare professionals need social support from their family members, relatives, and neighbors. Being devoid of that, support can result in anxiety and depression for healthcare professionals (41, 42). We predict that incentives such as economic support, constant supervision, sufficient protective equipment, and adequate workforce could motivate health workers to contribute more during pandemic situations. Unfortunately, Bangladeshi healthcare professionals are mostly deprived of these facilities. Some of the infected healthcare professionals of this study mentioned that though the government announced some financial incentives, they did not receive it in reality.

When comparing our finding with the SAARC countries, we see some striking similarities. These countries already have a vulnerable economy characterized by weak medical infrastructure that rarely managed to provide its people with sufficient medical care, at least providing the healthcare professionals with necessary psychological help (43). Inadequate PPEs, social stigma, and being victims of violence added extra psychological stress for healthcare professionals in the middle of their already hectic schedule (44). Besides, healthcare professionals from different age, gender, and socioeconomic background suffer from different psychological issues. A specialized set of interventions are required for healthcare professionals depending on their mental health condition (45). Although the National Health Policy of Bangladesh (2011) promises an adequate supply of logistics and manpower in government healthcare facilities, and coordination between different healthcare services-related departments (46), the reality is different. We observed that the lack of coordination and skilled manpower still remains a key problem affecting the healthcare services quality in the country, which corresponds to the existing literature (47, 48).

The spread of an epidemic can cause psychological trauma for healthcare professionals (10, 35). Therefore, effective coping strategies are required. Studies suggest that self-care, confidence, teamwork, and gathering among coworkers are some practical ways to alleviate mental pressure, work stress, and posttraumatic experiences amid emergencies of caregivers (49, 50) which was consistent with the results of this study. The stress theories also argue that the sustained response to stress for the healthcare professionals may lead to physical illness through proven pathophysiological ways (51, 52). We suggest as the pandemic prevails, healthcare professionals will face further physical and mental adversities; therefore, they will need a special attention to avoid this helpless situation.

## Strengths and Limitations

A strength of this study is using the exploratory qualitative inquiry to analyze what challenges the healthcare professionals in Bangladesh are facing and how they managed these adversities during the COVID-19 pandemic. However, the large volume of data was difficult to collect, analyze, and maintain. The researchers put a greater amount of effort and time to offset the limitations. We followed the consolidated criteria for reporting qualitative research (COREQ) checklist for our in-depth interviews and for reporting this study. The interviews were not restricted to specific questions and topics which helped producing rich and detailed information. We used the snowball sampling technique as we were unable to find a large number of healthcare professionals who were willing to allow us sufficient time and cooperation during the pandemic. Because of the hectic schedule of the healthcare workers, interviews had to be kept short in some cases. However, we managed to reach the desired number of participants required to complete the study. As qualitative research relies on the depth of information instead of the number participants, 15 healthcare professionals who participated in this study were enough for data saturation. Besides, executing a qualitative study through telephone interviews had its own limitations, although the researchers put their best effort to respond to the situation. We acknowledge that direct observation and methodological triangulation might have provided further insight into the topic.

## Conclusions

The present study explores the challenges faced by healthcare professionals during COVID-19 pandemic in Bangladesh. We found that insufficiency of medical staff as well as medical equipment was common and resulted in increased workload. Apart from this, shortage of PPE, fear of being infected, social exclusion, and mismanagement contributed further to put the healthcare professionals in adversity. Although the National Health Policy of Bangladesh (2011) recommends enhancing skilled manpower and logistic support, we found the actual scenario to be different. Especially during the COVID-19 outbreak that put the healthcare sector into unprecedented challenge, the promised coordination and support in the healthcare sector rather reflects a disparity between the policy and the practice. Despite the recently introduced National Infectious Diseases Act (2018), lack of a standardized COVID-19 protocol kept the medical professional under constant risk of infection and mental pressure. We conclude that the healthcare professionals need to be supported with adequate resources for both physical and mental health. While workloads need to be lessened, a proper coordination and access to information as promised in the National Health Policy during this public health emergency should be put in practice to ensure quality healthcare services.

## Data Availability Statement

The original contributions presented in the study are included in the article/Supplementary Material, further inquiries can be directed to the corresponding author.

## Ethics Statement

The studies involving human participants were reviewed and approved by Ethical Clearance Committee, Khulna University. Written informed consent for participation was not required for this study in accordance with the national legislation and the institutional requirements.

## Author Contributions

SR conceived the idea. SR, TY, TA, and MI analyzed the data. SR and TY drafted the manuscript. SR, SI, HG, and PW critically reviewed and approved the final version of the manuscript.

## Conflict of Interest

The authors declare that the research was conducted in the absence of any commercial or financial relationships that could be construed as a potential conflict of interest.

## Publisher's Note

All claims expressed in this article are solely those of the authors and do not necessarily represent those of their affiliated organizations, or those of the publisher, the editors and the reviewers. Any product that may be evaluated in this article, or claim that may be made by its manufacturer, is not guaranteed or endorsed by the publisher.
